# Successful Aging: Illness and Social Connections

**DOI:** 10.3390/geriatrics3010003

**Published:** 2018-01-18

**Authors:** Lisa F. Carver, Rob Beamish, Susan P. Phillips

**Affiliations:** 1 Department of Sociology, Queen’s University, Kingston, ON K7L 3N6, Canada; rob.beamish@queensu.ca; 2 Department of Medicine, Queen’s University, Kingston, ON K7L 3N6, Canada; susan.phillips@dfm.queensu.ca

**Keywords:** aging, geriatric health, successful aging, resilience, determinants of health, social connectedness

## Abstract

The objective of this study was to examine the role of social determinants of health: gender, income, education, housing, and social connections in successful aging in older adults aging with illness. Participants were 50 adults aged 65–90 years, all aging in place in their own home, and reporting at least one illness. This pilot study used non-probability sampling and employed both online and in-person interviews. The majority (82%) were aging “successfully” or “somewhat successfully” as reported on the single item successful aging scale and demonstrated by their scores on the Successful Aging Inventory (SAI). Correlations were not significant between SAI and gender, income, education, or housing. A significant negative correlation was found between SAI and community activity. However, there were significant positive correlations between SAI and religious activity and relationships. The regression model was a linear combination of participants’ community and religious activity and relationships. The majority of older adults aging with illness consider themselves to be aging successfully, but their scores are influenced by relationships with others as well as religious and community activity. Frequent community activity had a suppressor effect on successful aging.

## 1. Introduction

Aging is an experience that can be endured or embraced. Successful aging focuses on embracing the journey and “adding life to years not years to life” [1]. It includes life satisfaction and connection to loved ones [2]. In our previous research, we identified that success in aging is associated with self-reported health, resilience, and engagement [3]. This study focused on whether “social determinants of health,” such as gender, social connectedness, income insufficiency, lack of education, and housing stability influence successful aging with illness [4]. People with higher socioeconomic status (SES) typically have higher levels of education and income, better health, and are more likely to engage in health protective behaviours (e.g., engage in health screening, exercise, and healthy eating habits); lower SES is normally associated with lower income and education levels and is often associated with illness and chronic disease [5]. 

Social determinants of health and illness have been associated with the health outcomes that manifest in later life [4]. Given that the majority of adults over 65 years old are living with at least one illness, we considered that social determinants of health may hold the key to enhancing successful aging among those over 65 years of age. This study explores the research question: Do the social determinants of health as income, education, social connectedness, gender, and housing stability play a role in successful aging with illness? A cross-sectional, observational design was used, with non-probability sampling methods self-selection and snowball sampling [6]. Based on the literature reviewed, we hypothesized that successful aging would be influenced by these social determinants of health.

## 2. Method

### 2.1. Participants

Ethics clearance was obtained from The Queen’s University Health Sciences and Affiliated Teaching Hospitals Research Ethics Board (HSREB ROMEO/TRAQ#: 6013128). All participants were between 65 and 90 years old and reported at least one illness. Table 1 provides the descriptive statistics for the 50 participants who were, for the most part, residents of Ontario, Canada (86%). An online version of the questionnaire allowed for the inclusion of older adults from anywhere, and 6% of participants were from other locations in the USA and Canada. Eight percent were in unknown locations (participants had the option of not providing their location). 

### 2.2. Measures

**Gender.** Given the need to keep the interview as short as possible, gender was measured using the self-report gender measure [7]. The question was worded as “Most of the time would you say you are:” Answer choices were as follows: “very feminine”, “mostly feminine”, “a mix of masculine and feminine (also referred to as androgynous)”, “neither masculine or feminine (also referred to as gender neutral)”, “mostly masculine”, “very masculine” or “other”.

**Social connectedness.** Social connectedness was assessed by asking participants about their frequency of community activities. The following two questions were asked: “do you go to a community centre, recreation centre, senior centre, or professional association?” and “do you participate in religious activities such as attending services, committees, and/or choirs?” The possible answers were on a five-point scale. Answer choices were never (scored as 1), once a year (scored as 2), once a month (scored as 3), once a week (scored as 4), and almost every day (scored as 5).

A third question was also used for social connectedness: “Are you happy with the relationships that you have with your friends and/or family?” The answer choices for this question were as follows: very happy (scored as 5), happy (scored as 4), neutral (scored as 3), unhappy (scored as 2), and very unhappy (scored as 1).

**Self-Rated Successful Aging (SR-SA).** Self-rated successful aging is a common tool used instead of a questionnaire to determine participants’ opinions of their own aging [8,9,10]. We constructed a question to assess self-rated successful aging, and this question was, simply, “How successfully are you aging (your physical, mental, and social well-being as you have become older)?” Self-rated success was recorded on a 5-point Likert scale. The available choices were as follows: “I have aged successfully” (scored as 5), “I have aged somewhat successfully” (scored as 4), “I don’t consider my aging successful or unsuccessful” (scored as 3), “I am aging somewhat unsuccessfully” (scored as 2), and “I have not aged successfully” (scored as 1).

**Successful Aging Inventory (SAI).** The SAI [11,12] has 20 items, uses a Likert format from 0 to 4, and takes approximately 10 min to complete. Respondents indicate their level of agreement with statements or the extent to which they believe the statement applies to them. There are five components: intrapsychic and functional performance, coping mechanisms, existential being, introspective gerotranscendence, and retrospective gerotranscendence. Scoring of the SAI ranges from 0 to 80 with higher scores reflecting more successful aging. Previous research has recorded SAI means of 62–65, with minimum to maximum scores ranging from 18 to 80 and standard deviations of 9.32–11.23 [13]. The SAI has demonstrated good convergent validity: significant positive correlations with measures of life satisfaction and significant negative correlations with indicators of depression and good reliability. The associated Cronbach’s alphas from .86 to 0.91 indicate solid internal consistency [12].

### 2.3. Statistical Analysis

Quantitative analysis was used to identify relationships between successful aging (as measured using the Successful Aging Inventory (SAI)) and sex, gender, income adequacy, education level, and social connectedness (community activity, religious activity, and quality of relationships with family/friends).

## 3. Results

Lung and heart disease were each reported 12 times. Cancer was present in 9 people. Eight people had diabetes or pre-diabetes and three had stroke related illness. Fifteen people reported other illnesses (e.g., arthritis and kidney disease). Eight people also reported having lost 5 kg or more unintentionally in the past year. Almost half (44%) of the participants were currently married, 32% were widowed, 22% were separated or divorced, and 2% were single. All described their housing as stable and were aging in place in their own home or apartment. Forty-eight percent lived alone, while the rest (52%) lived with at least one other person. An independent sample t-test did not reveal significant differences in SAI for those who live alone versus those who live with someone else (*t*(44) = −1.275, *p* = 0.209).

Participants were given the opportunity to identify their sex as “male” (32%), “female” (68%) or “other.” No one chose the “other” category. Gender, as shown in Table 1, was significantly correlated with education (*r* = 0.395, *p* < 0.01), community activity (*r* = 0.328, *p* < 0.05), and sex (*r* = 0.774, *p* < 0.001). Gender was not correlated with the SAI. The majority of participants (74%) had a college diploma, undergraduate degree, graduate degree, or medical degree (Table 1). Most (93%) reported that their income was adequate. Neither education nor income was significantly correlated with SAI. Variables that were not significantly correlated with SAI were excluded from regression analysis. 

**Successful Aging.** Despite the presence of illness, the majority of these participants (82%) reported successful aging on the SR-SA (Table 1). No one self-reported “unsuccessful aging.” There was a significant positive correlation (*r* = 0.422, *n* = 46, *p* = 0.003) between the SR-SA and SAI. The majority of the social determinants of health (income, education, and gender) were not significantly correlated with SAI. 

The SAI was normally distributed (Shapiro-Wilk test, *p* = 0.831). Descriptive statistics for the SAI and the social connectedness variables used in the regression analysis are shown in Table 2. The SAI was used as the outcome in the regression model.

**Regression analysis:** Together the social connectedness variables predicted successful aging as measured using the SAI (*F*(3, 42) = 7.226, *p* = 0.001, R^2^ = 0.340). As shown in Table 3, each independent variable contributed significantly to the model: (religious activity: *β* = 0.302, *p* = 0.021; relationships: *β* = 0.308, *p* = 0.019; community activity: *β*= −0.367, *p* = 0.006). The negative weight for the standardized Beta coefficient for community activity indicates that those participants with higher community activity scores have lower successful aging scores.

## 4. Discussion

Despite the age-old themes in this research project, the exploration of successful aging with illness is important [3]. All of the participants reported at least one illness and stable housing, living in their own home or apartment. An ageist notion we grappled with was that surveying older adults online would be problematic as this population is not considered by many as Internet-savvy. However, 81% of participants completed the survey online, indicating that this is a viable way to reach out to people over 65 years old. Factors that had no significant impact on aging successfully in this cohort were online or in-person participation, gender, income adequacy, education level, living alone, and location of residence. The mean score on the successful aging inventory (SAI) was higher than that found by others [13].

We hypothesized that social connectedness (community activity, religious activity, and relationships) might be associated with successful aging. All of these variables had a significant correlation with successful aging, but the results suggest that the relationship is complex. A key finding in this study was that community activity had a suppressor effect: those who were more frequently engaged in community considered themselves to be less successful in aging; conversely, people who were less often involved in community activities reported higher levels of successful aging. It may be that people who seek out community activities at least once a week do so because they are aging less successfully. It could also reflect the impact of volunteer fatigue, found in other research [14]. Finally, the theory of gerotranscendence may apply, suggesting that withdrawal from community activity is a healthy choice and reflects consolidation of activities in the face of dwindling time. Certainly participants themselves reinforced the importance of family and close friends, saying “what is important in life is faith, unconditional love and relationships and humor” and “we learn that everyone has something to offer and that most people do the best that they are able in life, given the circumstances of their upbringing.” Irrespective of the reasons for these results, community activities play a role in successful aging. 

## 5. Limitations

We recognize that there are a number of limitations to this study: the use of a cross-sectional design can identify associations in the data but is unable to determine causality [15]; non-probability purposive sampling introduces a selection bias and precludes generalization of findings to the population at large [16,17]; self-report measures rely on the honest and accurate reflection of participants; and the exclusion of institutionalized participants means that levels of successful aging may reflect a higher functioning sample [18]. Future research could be conducted with a larger sample, disaggregated by sex/gender and with stratification by age groups, (e.g., 65–79 and 80 or older) [19,20,21,22]. In this study, there were too few participants to have the statistical power to do disaggregated analyses.

## 6. Conclusions

Given that community activities reflect social connectedness, the possibility that they are playing a suppressor role in successful aging with illness is vital to explore in greater depth. Very little successful aging research has focused entirely on those aging with illness; it may be that the experience of successful aging *with illness* involves different constructs and determinants than those seen among people aging *without illness*. The reality is that those aging with illness are actively involved in their own care, as seen in this quote by a participant:

My skin is thinning. Bruises and marks are annoying. Nothing you can do about it. You have to take an interest in your body and figure out what you need and what works for you. The doctor can’t tell you. You have to do it yourself. Get up and do exercises, check it out with your doctor. Stop the smoking and over-eating. The other thing is you have to keep the mind active.

They engage with their treatment but are not defined by their illnesses. Quite often, among these participants, they do not even consider themselves “sick”; rather, they are people who happen to have an illness. We encourage researchers, policy makers, and service providers to develop resources for older adults that are accessible and include those with illness and disability. 

Our goal in this research was to better understand the influence of social determinants of health among those aging with illness and to generate awareness and knowledge of the positive aspects of aging with an illness among older adults. We hope that the understanding that people aging with illness often feel successful in their aging may result in greater inclusion of older adults with illness in the generation of policy to support success in aging and in practice changes.

## 7. Key Points

- The majority of participants aging with illness considered themselves to be aging successfully.
- Social connectedness variables community activity, religious activity, and relationships were predictive of successful aging in people aging with illness.
- Engagement in community activities had a suppressor effect on successful aging scores.

## Figures and Tables

**Table 1:** Description of participants.

	Range	Mean
Age (years) (*n* = 50)	65–90	74.76
	***n***	**%**
**Sex**		
Female	34	68
Male	16	32
**Gender**		
Very feminine	12	23
Mostly feminine	16	30
Mix of masculine and feminine	7	13
Mostly masculine	8	17
Very masculine	6	15
*Missing data*	*1*	*2*
**Location**		
Ontario	43	86
USA	1	2
Nova Scotia	1	2
Quebec	1	2
Unknown	4	8
**Survey Type**		
Online	40	80
In-person	10	20
**Relationship status**		
Single	1	2
Married/common-law	22	44
Widow/widower	16	32
Separated/divorced	11	22
**Income Adequacy**		
Not very well	3	6
Reasonably well	27	54
Very well	20	40
**Education level**		
Completed grade 8	4	8
Completed high school	10	20
College	12	24
University (undergraduate)	13	26
University (graduate)	11	22
**Self-report successful Aging (SR-SA)**		
Successful	23	46
Somewhat successful	18	36
Neither successful/unsuccessful	8	16
Somewhat unsuccessful	1	2

Percentages subject to rounding. May not sum to 100%.

**Table 2:** Summary of social determinants of health in the regression analysis.

	Min. to Max.	Mean	SD
Successful aging inventory (*n* = 46)	*53–80*	68.19	6.10
**Social Connectedness (*n* = 50)**			
Community activities	Frequency	Percent	
Almost every day	6	12	
Once a week	19	38	
Once a month	8	16	
Once a year	3	6	
Never	14	28	
**Relationships**	Frequency	Percent	
Very happy	27	54	
Happy	19	38	
Neutral	1	2	
Unhappy	2	4	
Very Unhappy	1	2	
**Religious activities**	Frequency	Percent	
Almost every day	3	6	
Once a week	12	24	
Once a month	3	6	
Once a year	8	16	
Never	24	48	

**Table 3:** Summary of regression analysis for variables predicting successful aging.

	Model 1		
**Variable**	*B*	*SE B*	***β***
**Community activity**	−1.552	0.531	−0.367 **
**Religious**	1.269	0.527	0.302 *
**Relationships**	2.129	0.869	0.308 *
***R*^2^**	**0.340**		

*Note:* * *p* < 0.05. ** *p* < 0.01.
